# Estradiol Alters the Virulence Traits of Uropathogenic *Escherichia coli*

**DOI:** 10.3389/fmicb.2021.682626

**Published:** 2021-07-20

**Authors:** Ulrik Engelsöy, Maria A. Svensson, Isak Demirel

**Affiliations:** ^1^School of Medical Sciences, Örebro University, Örebro, Sweden; ^2^Department of Research and Education, Faculty of Medicine and Health, Örebro University, Örebro, Sweden; ^3^Faculty of Medicine and Health, iRiSC–Inflammatory Response and Infection Susceptibility Centre, Örebro University, Örebro, Sweden

**Keywords:** uropathogenic *Escherichia coli*, estradiol, growth, virulence, cross-kingdom interaction

## Abstract

Uropathogenic *Escherichia coli* (UPEC) is the most common bacteria to cause urinary tract infection (UTI). Postmenopausal women have an increased risk of recurrent UTI. This is partly explained by estrogenic effects on host defenses against UTI. Current research is mostly focused on how UPEC affects host factors, but not so much is known about how host factors like hormones affect UPEC virulence. The aim of the present study was to investigate the impact of estradiol exposure on the virulence of UPEC. We found that a postmenopausal concentration of estradiol increased CFT073 growth and biofilm formation, but not the premenopausal concentrations. Real-time qPCR showed that estradiol altered the expression of genes associated with the iron acquisition system and metabolic pathways in CFT073. We also found that estradiol in a dose-dependent manner increased the expression of *fimH* and *papC* adhesins and increased colonization and invasion of bladder epithelial cells. The premenopausal concentration of estradiol also suppressed cytokine release from bladder epithelial cells. Additionally, we also showed using a *Caenorhabditis elegans* killing assay that estradiol increased the survival of CFT073-infected *C. elegans* worms. Taken together, our findings show that estradiol has the ability to alter the virulence traits of UPEC.

## Introduction

Urinary tract infection (UTI), the majority caused by uropathogenic *Escherichia coli* (*E. coli*), is one of the most widespread infections in humans. Approximately 50% of all women will suffer from one UTI during their lifetime. Furthermore, women with a history of UTI have an increased risk of recurrency within 3–4 months (Flores-Mireles et al., 2015). We know today that estrogen is very important for the fight of the host against UTI (Lüthje et al., 2013, 2014). The female body becomes more prone to UTI after menopause because lower estrogen levels lead to changes all along the urinary tract. Reduced levels of estrogen in postmenopausal women are associated with decreased proliferation of bladder epithelium (Blakeman et al., 2001) and lower levels of antimicrobial peptides (AMPs) (Lüthje et al., 2014). In addition, lower estrogen levels induce vaginal mucosa atrophy, leading to a decreased number of lactobacilli and, hence, a higher pH. The reduced number of lactobacilli and the higher pH are associated with an increased risk of urinary tract colonization by UPEC (Gupta et al., 1998). In the 1990s, several studies were conducted focusing on how to reduce recurrent UTI in menopausal women (Kjaergaard et al., 1990; Cardozo et al., 1998; Eriksen, 1999). One study investigated the use of topical estrogen cream vs. prophylactic antibiotics in postmenopausal females as a treatment against recurrency. The study found that the control group without any treatment experienced an average of 5.9 UTI episodes per year. With estrogen treatment, the average dropped to 0.5 episodes per year compared with 0.8 episodes per year with an antibiotic (Raz and Stamm, 1993). Hence, vaginal estrogen therapy was shown to be able to reduce the number of episodes of UTI in menopausal women.

Most of the research conducted today in the field of host–pathogen interaction is focused on elucidating how pathogens, with their respective virulence factors, successfully modulate or evade the immune responses to cause infections. However, less is known about how host immune factors like cytokines and hormones are affecting the virulence of UPEC by cross-kingdom interaction. The majority of the present research conducted on UTI and estrogen has focused on its effects on the host and analysis of risk factors. Wang et al. (2013) have shown that mice with low estrogen levels have higher levels of bacteriuria compared with controls in a UTI model. The effect of host factors (e.g., hormones and proinflammatory cytokines) on bacteria is a relatively unexplored field. What has been shown is that cytokines can bind to bacterial DNA and alter gene expression in *Neisseria meningitidis* (Mahdavi et al., 2013). We have also shown that proinflammatory cytokines alter UPEC virulence, leading to significantly decreased survival of *C. elegans* worms (Engelsöy et al., 2019). Estrogen has been shown to enhance the growth and survival of several Gram-negative bacteria. The interaction of estradiol with *Pseudomonas aeruginosa* results in an enhancement of its virulent mucoid biofilm phenotype (Chotirmall et al., 2012). The mechanism for this shift appears related to a *P. aeruginosa* constitutive cytosolic estrogen-binding protein (Rowland et al., 1992). In *Chlamydia trachomatis*, estradiol downregulates a significant portion of genes involved in nucleotide metabolism and fatty acid biosynthesis and upregulates genes associated with the chlamydial stress response (Amirshahi et al., 2011). These studies strengthen the notion that estrogen has a direct effect on bacterial virulence. A recent *in vitro* study investigated the effects of estrogen on *E. coli* growth and gene expression. They showed that estrogen increased the growth of *E. coli* (Gümüş et al., 2019). Hence, we know that there is a strong clinical association between estradiol levels and the development of a UPEC-mediated UTI. However, we do not know if the direct effects of estradiol on UPEC virulence could partially explain the association between estrogen levels and UPEC-mediated UTI. The aim of this study was to investigate the impact of estrogen exposure on the virulence of UPEC.

## Materials and Methods

### Cell and Bacterial Culture

CFT073 is a UPEC strain isolated from a patient with pyelonephritis, which is fully genome sequenced (Welch et al., 2002). The bacteria were kept on a tryptic soy agar plate (Becton Dickinson, Franklin Lakes, NJ, United States). CFT073 containing an enhanced green fluorescent protein (eGFP) expressing pLMB449 plasmid (Karunakaran et al., 2005) (kind gift from Professor Philip Poole at the University of Oxford, Oxford, United Kingdom) was used for the colonization experiments.

The bladder epithelial cell line 5,637 is a commercial cell line acquired from the American Type Culture Collection (Manassas, VA, United States). The cells were grown in Dulbecco’s modified Eagle’s medium (DMEM) (Lonza, Basel, Switzerland) with 10% fetal bovine serum (FBS), 2 mM L-glutamine, 1 mM non-essential amino acids (Thermo Fisher Scientific, Waltham, MA, United States) and incubated at 37°C and 5% CO_2_ atmosphere. The culture medium was changed during the experiments to DMEM with 2% FBS, 1 mM non-essential amino acids, and 2 mM L-glutamine.

### Growth Assessment

The UPEC strain CFT073 was grown in Lysogeny broth (Becton Dickinson) overnight on a shaker at 37°C prior to the growth assay. CFT073 (1 × 10^6^ CFU/ml) was then grown in minimal salt medium [MSM, 0.3% KH_2_PO_4_, 1.3% (wt/vol) Na_2_HPO_4_, 0.05% NaCl, and 0.1% NH_4_Cl supplemented with 20 mM glucose, 2 mM MgSO_4_, 100 mM CaCl_2_, and 0.25% casamino acids] with or without the presence of 17β-estradiol [5 pg/ml, 10 pg/ml, 100 pg/ml, 300 pg/ml, 1 ng/ml, and 10 ng/ml (E2758, Sigma-Aldrich, St. Louis, MO, United States)] in a 96-well plate. The 96-well plate was then incubated at 37°C and the optical density (600 nm) was measured every 10 min using a spectrophotometer (Cytation 3, Biotek Inc., Winooski, VT, United States).

### Biofilm and Endotoxin Measurement

After the growth assay, the same 96-well plate was used for evaluating biofilm formation after 24 h. The wells were washed with sterile RO water three times. Crystal violet (0.1%, Thermo Fisher Scientific) was added to the wells to stain the biofilm. Excess crystal violet was washed away with RO water and the plate was left to dry overnight. Ethanol (95%) was then used to dissolve the crystal violet and the solution was transferred to a new plate and the absorbance (540 nm) was measured by a spectrophotometer (Cytation 3).

CFT073 was grown in MSM in the presence or absence of estradiol (5 and 300 pg/ml) statically at 37°C for 24 h. After 24 h of stimulation, the bacterial supernatants were centrifuged for 5 min at 5,000×*g* and the endotoxin levels were analyzed using Pierce LAL chromogenic endotoxin quantitation kit (Thermo Fisher Scientific) according to the instructions of the manufacturer.

### RNA Isolation, cDNA Generation, and Quantitative Polymerase Chain Reaction

CFT073 (1 × 10^6^ CFU/ml) was grown in MSM with or without estradiol (5 and 300 pg/ml) statically at 37°C for 6 or 24 h. RNAlater (Sigma-Aldrich) was used before RNA isolation to stabilize the mRNA in CFT073. The E.Z.N.A^®^ Total RNA Kit I (Omega Bio-tek, Inc., Norcross, GA, United States) was used according to the instructions of the manufacturer for the isolation of total RNA. DNase digestion (TURBO DNase, Life Technologies, Waltham, MA, United States) was conducted according to the instructions of the manufacturer to minimize DNA contamination. RNA concentration and purity were measured with a spectrophotometer (Nano Drop 2000, Wilmington, NC, United States) before cDNA synthesis. The cDNA synthesis was performed with 100 ng total RNA using the High-Capacity cDNA Reverse Transcription Kit (Applied Biosystems, Foster City, CA, United States). For the RT-qPCR, 5 ng cDNA and 250 nM of primer (Table 1) (Eurofins MWG Synthesis GmbH, Ebersberg, Munich, Germany) were used with Maxima SYBR Green qPCR Master Mix (Thermo Fisher Scientific). A CFX96 Touch^TM^ Real-Time PCR Detection System (Bio-Rad, Hercules, CA, United States) was used for the amplification using the following protocol for 40 cycles: denaturation at 95°C for 15 s, annealing at 60°C for 30 s, and extension at 72°C for 30 s. A dissociation curve between 60 and 95°C was also done after the qPCR. CT values were obtained and the ΔΔCt method (2^–ΔΔCt^) was used to calculate the fold difference between groups. The results were normalized to the endogenous control glyceraldehyde 3-phosphate dehydrogenase A (*gapA*).

**TABLE 1:** Primers used in the real-time qPCR.

Gene symbol	Description	Oligonucleotide sequences (5′–3′)
*iutA*	Siderophore receptor	*F*: AAAGAGCTGAAAGACGCACTGG *R*: TGTCGGAACGTGAAGAGTTGAG
*iroN*	Siderophore receptor	*F*: ATTACCAAACGTCCCACCAACG *R*: AAACGCGTGGTAAGAGCATCAC
*iha*	Siderophore receptor	*F*: TGCGAATAACCACTCTGGCTTC *R*: TAATCACAGAAACACTGGCGGC
*chuA*	Heme receptor	*F*: AAGGCGTTGCCCAATACCAGAGTA *R*: TATTCCGATCGCTCACAGTGGCTT
*FimH*	Adhesin subunit of type 1 fimbriae	*F*: GTGCCAATTCCTCTTACCGTT *R*: TGGAATAATCGTACCGTTGCG
*papC*	Enables P-fimbriae assembly	*F*: GTGGCAGTATGAGTAATGACCGTTA *R*: ATATCCTTTCTGCAGGGATGCAATA
*pgi*	Glycolytic enzyme	*F*: CTCTGGCGAGAAGATCAACC *R*: TCACCGGAAATAATCGCTTC
*ppsA*	Gluconeogenetic enzyme	*F*: GCAAAACAGGCCGTACAAAT *R*: CAGCGTATAACGCTCCATGA
*frdA*	Anaerobic respiratory enzyme	*F*: CAACACCGACCTGCTCTACA *R*: GCGGCAGCGTAGTAATCTTC
*gapA*	Endogenous control (glycolytic enzyme)	*F*: AAGTTGGTGTTGACGTTG *R*: AGCGCCTTTAACGAACATCG

### Colonization Assay

CFT073 (harboring an enhanced GFP-expressing plasmid, eGFP) was grown in MSM (with gentamicin) in the presence of absence of estradiol (5 and 300 pg/ml) statically at 37°C for 24 h. Estradiol was washed away from the bacteria with phosphate-buffered saline (PBS) and the bladder epithelial cell line 5,637 (50,000 cells) was infected with the respective treatment at multiplicity of infection (MOI) of 10 for 4 h at 37°C and 5% CO_2_ to measure bacterial colonization (adherent and intracellular bacteria). Then, the cells were washed with PBS 10 times and the eGFP-expressing CFT073 was quantified with the Cytation 3 plate reader. Colonization is presented as % mean fluorescence intensity (MFI) of CFT073.

### Invasion Assay

CFT073 was grown in MSM in the presence or absence of estradiol (5 and 300 pg/ml) statically at 37°C for 24 h. Estradiol was washed away from the bacteria with PBS and the bladder epithelial cell line 5,637 (250,000 cells) was infected with the respective treatment at MOI of 100 for 2 h at 37°C and 5% CO_2_. Then, the cells were washed with PBS 10 times. DMEM supplemented with 2% FBS and 100 μg/ml gentamicin was then added to the cells to kill remaining extracellular bacteria during 2 h. The plate was then washed again three times and the cells were lysed with 0.1% Triton X-100 in PBS (with calcium chloride 100 mg/L and magnesium chloride 100 mg/L) for 10 min under gentle rotation. Finally, the bacteria were plated on TSA plates and incubated overnight at 37°C, and the colonies were counted the next morning.

### Cytokine Release and Viability Assay

CFT073 was grown in MSM in the presence of absence of estradiol (5 and 300 pg/ml) statically at 37°C for 24 h. Estradiol was washed away from the bacteria with PBS and the bladder epithelial cell line 5,637 (50,000 cells) was infected with the respective treatment at MOI of 10 for 6 h at 37°C and 5% CO_2_. Supernatants were collected after the infection and centrifuged for 5 min at 5,000×*g* and stored at –80°C. An enzyme-linked immunosorbent assay (ELISA) was performed to measure IL-1β and IL-8 release from the 5,637 cells. The cytokine was measured with the IL-1β and IL-8 kits (ELISA MAX Deluxe Sets, BioLegend, San Diego, CA, United States) according to the instructions of the kit. Cell viability after infection was assessed by Pierce LDH cytotoxicity assay (Thermo Fisher Scientific) according to the instructions of the kit.

### *Caenorhabditis elegans* Killing Assay

The *C. elegans* wild-type Bristol strain N2 (Caenorhabditis Genetics Center, University of Minnesota, United States) (Brenner, 1974) was maintained on nematode growth medium plates seeded with *E. coli* OP50 (Brenner, 1974) (Caenorhabditis Genetics Center) at 21°C. Prior to the experiments, *C. elegans* were synchronized (0.25 M NaOH, 1% HOCl) and maintained on nematode growth medium plates for 48 h at 21°C to reach the L4 stage. CFT073 was grown in MSM in the presence or absence of estradiol (5 and 300 pg/ml) statically at 37°C for 24 h. Estradiol was washed away from the bacteria and CFT073 (5 × 10^8^ CFU/ml) was transferred to a 96-well plate. The L4 worms were washed with M9 buffer and 10 worms were then transferred to the respective well of bacteria that had been grown in the presence or absence of estradiol. After the addition of the worms to the wells, *C. elegans* and the bacteria were incubated together for 10 h at 21°C. The viability of the worms was evaluated every hour. A worm was considered dead when it failed to respond to touch. Dead worms were also visualized with 1 μM SYTOX Green (Thermo Fisher Scientific) using a spectrophotometer (Cytation 3) (Gill et al., 2003).

### Statistical Methods

Student’s unpaired *t*-test was used to analyze the difference between stimulated CFT073 and unstimulated CFT073. Data are expressed as mean ± SEM. Results were considered statistically significant at *p* < 0.05. *n* is equal to the number of independent biological experiments.

## Results

### Estradiol Induces Increased UPEC Growth

**FIGURE 1:**
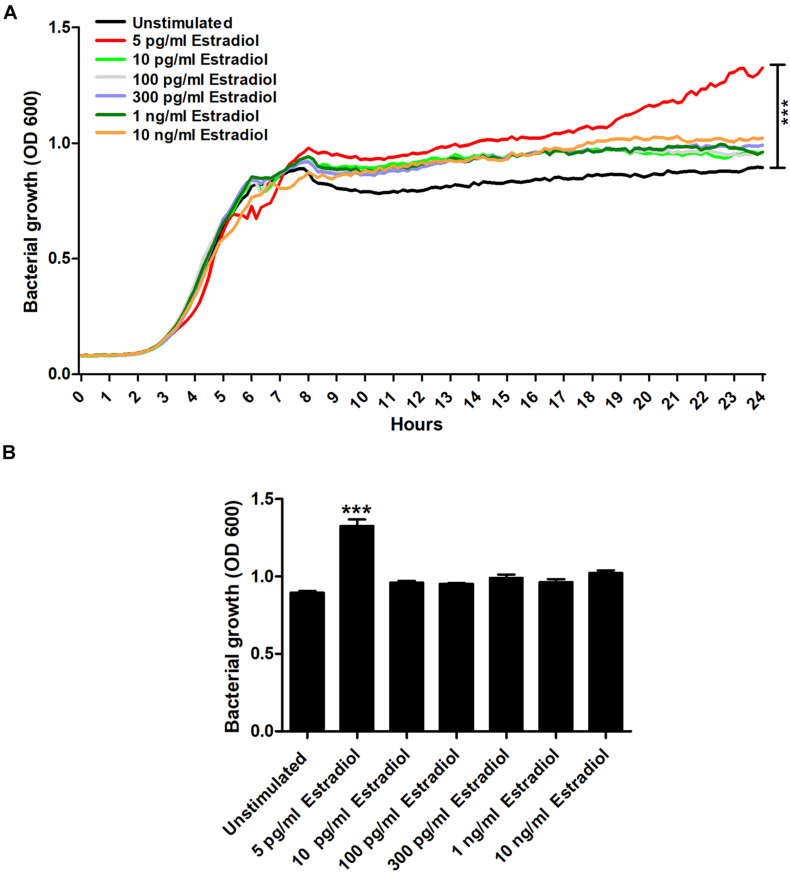
CFT073 growth with or without the presence of estradiol (5 pg/ml, 10 pg/ml, 100 pg/ml, 300 pg/ml, 1 ng/ml, and 10 ng/ml) during 24 h **(A)** and at 24 h **(B)**. Data are presented as mean **(A)** and mean ± SEM **(B)** of *n* = 3 independent experiments. The asterisk distinguishes statistical significance: ***p < 0.001 vs. unstimulated CFT073.

We began by evaluating the effects estradiol had on the growth of CFT073. We found that 5 pg/ml of estradiol significantly increased the growth of CFT073 compared with unstimulated CFT073 (Figure 1A). Statistical significance (*p* < 0.05) was reached from 8 h onward for 5 pg/ml estradiol. The biggest growth difference was observed between 5 pg/ml estradiol and unstimulated CFT073 after 24 h (Figure 1B). However, the higher concentrations of estradiol did not induce a significant growth increase compared with unstimulated CFT073 (Figures 1A,B).

### Biofilm Formation and Endotoxin Release

**FIGURE 2:**
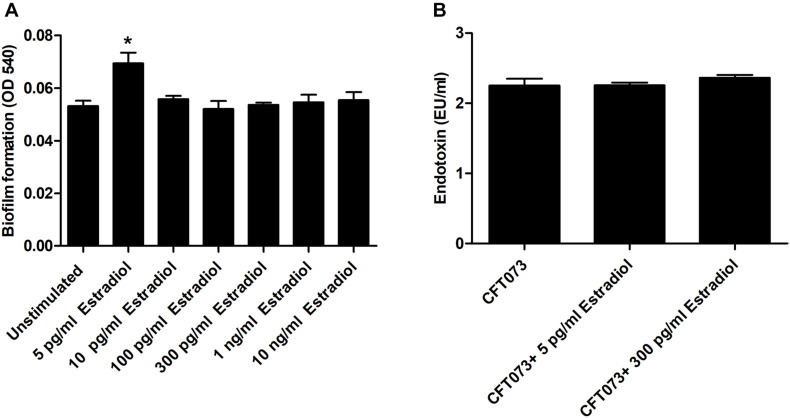
Biofilm formation **(A)** and endotoxin release **(B)** in the presence or absence of estradiol after 24 h. Data are presented as mean ± SEM of *n* = 3 independent experiments. The asterisk distinguishes statistical significance: *p < 0.05 vs. unstimulated CFT073.

We continued by investigating the effects of estradiol on biofilm formation and endotoxin release. The biofilm formation was significantly increased by 5 pg/ml estradiol compared with unstimulated CFT073 after 24 h. The higher concentrations of estradiol did not differ from unstimulated CFT073 in their ability to induce biofilm formation (Figure 2A). When looking at endotoxin levels, we found no significant changes induced by estradiol compared with unstimulated CFT073 (Figure 2B).

### Estradiol Alters the Expression Levels of Genes Encoding Different Metabolic Pathways, Fimbriae, and Iron Acquisition Systems

**FIGURE 3:**
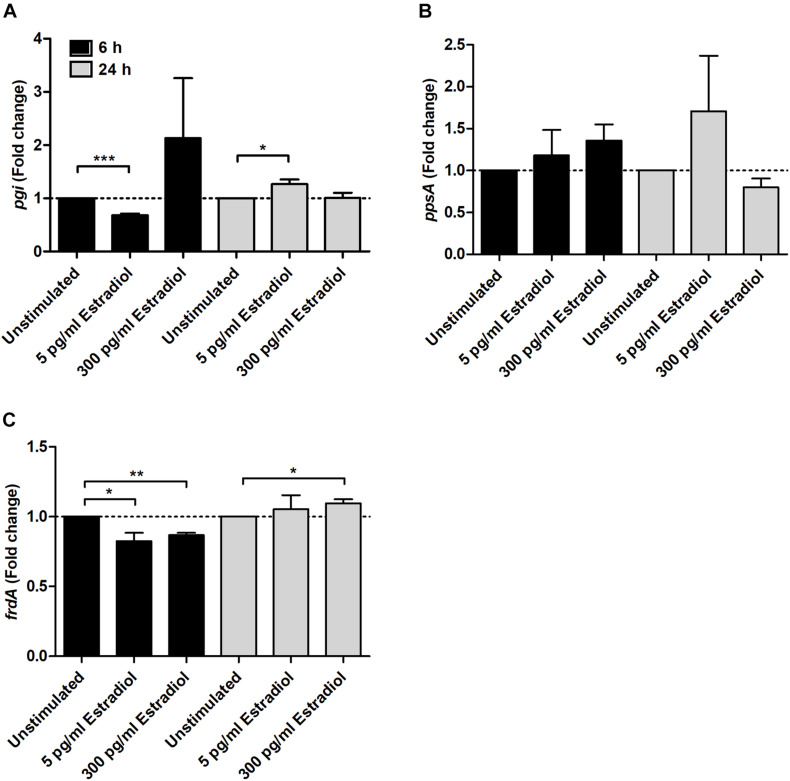
Real-time qPCR analysis of *pgi* **(A)**, *ppsA* **(B)**, and *frdA* **(C)** gene expression in the presence or absence of estradiol (5 and 300 pg/ml) at 6 and 24 h. Data are presented as mean ± SEM of *n* = 3 independent experiments. The asterisks distinguish statistical significance: *p < 0.05; **p < 0.01; ***p < 0.001 vs. unstimulated CFT073.

We investigated the effects of estradiol on virulence-associated genes in UPEC. We found that the gene expression related to energy metabolism was altered. Estradiol at 5 pg/ml significantly changed the expression of *pgi* at both 6 and 24 h compared with unstimulated CFT073 (Figure 3A). At 6 h, the expression was decreased, and at 24 h, an increase was observed. The expression of *ppsA* showed no significant changes, but a possible trend toward an increased expression was found compared with unstimulated CFT073 (Figure 3B). As for *frdA*, the expression was significantly decreased at 6 h by 5 and 300 pg/ml estradiol. At 24 h, a slight increased expression induced by 300 pg/ml estradiol was seen (Figure 3C).

**FIGURE 4:**
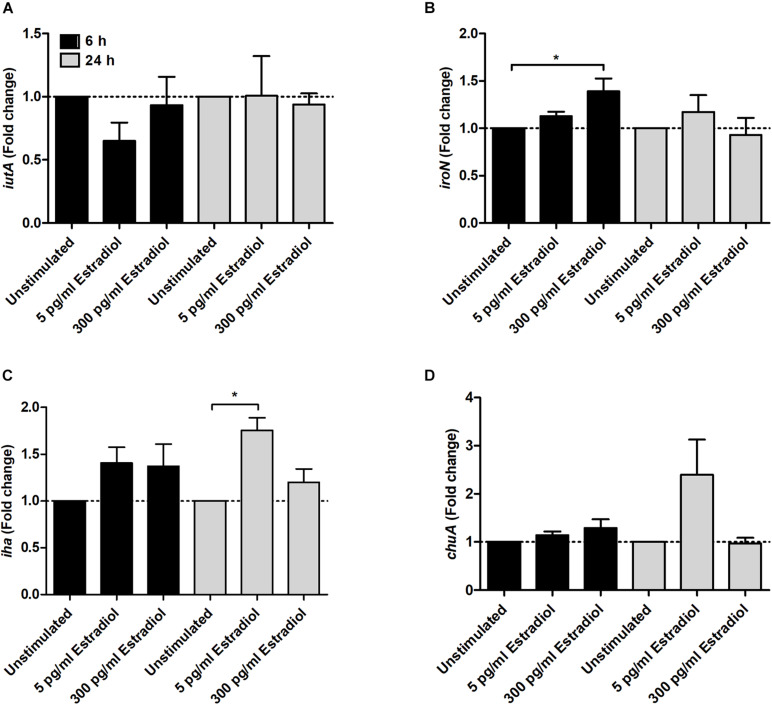
Real-time qPCR analysis of *iutA* **(A)**, *iroN* **(B)**, *iha* **(C)**, and *chuA* **(D)** gene expression in the presence or absence of estradiol (5 and 300 pg/ml) at 6 and 24 h. Data are presented as mean ± SEM of *n* = 3 independent experiments. The asterisks distinguish statistical significance: *p < 0.05 vs. unstimulated CFT073.

**FIGURE 5:**
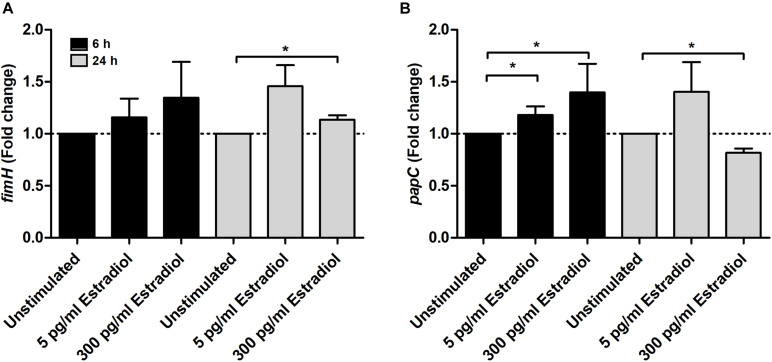
Real-time qPCR analysis of *fimH* **(A)** and *papC* **(B)** gene expression in the presence or absence of estradiol (5 and 300 pg/ml) at 6 and 24 h. Data are presented as mean ± SEM of *n* = 3 independent experiments. The asterisks distinguish statistical significance: *p < 0.05 vs. unstimulated CFT073.

Regarding iron acquisition-associated genes, we found that 300 pg/ml estradiol increased the expression of *iroN* at 6 h (Figure 4B) and 5 pg/ml estradiol increased the expression of *iha* at 24 h (Figure 4C) compared with unstimulated CFT073. However, estradiol did not induce any significant changes in the expression of the *iutA* (Figure 4A) or *ChuA* genes (Figure 4D).

For adhesion-associated genes, the expression of *papC* was significantly increased at 6 h by both 5 and 300 pg/ml estradiol compared with unstimulated CFT073. After 24 h, *papC* expression was significantly decreased by 300 pg/ml estradiol (Figure 5B). The *fimH* expression was increased by estradiol at 6 h but significance was not reached. However, the expression of *fimH* was significantly increased by 300 pg/ml estradiol at 24 h compared with unstimulated CFT073 (Figure 5A).

### Colonization and Invasion of Bladder Epithelial Cells

**FIGURE 6:**
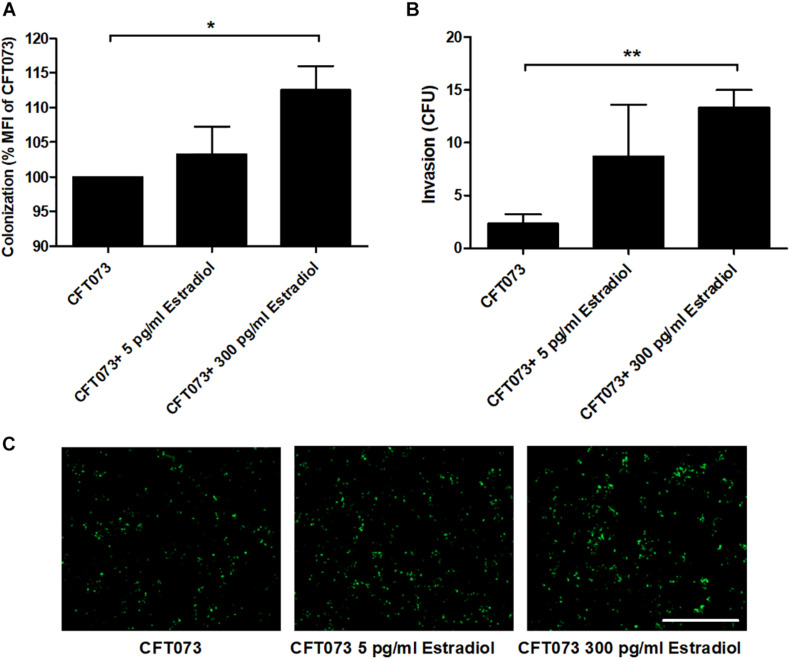
CFT073 colonization (**A**,**C**, 4 h) or invasion (**B**, 2 h) of bladder epithelial cells after CFT073 pretreated with or without estradiol (5 and 300 pg/ml). CFT073 (harboring a GFP-expressing plasmid) colonization was quantified as mean fluorescence intensity (MFI) **(A)** and imaged **(C)**. Data are presented as mean ± SEM of *n* = 3 independent experiments. The asterisk distinguishes statistical significance: *p < 0.05, **p < 0.01 vs. unstimulated CFT073. Scale bar: 200 μm.

We proceeded with evaluating the effects of estradiol on the ability of UPEC to colonize (adhere and invade) and invade human bladder epithelial cells. Bacterial colonization, at MOI 10, seems to increase with estradiol-stimulated CFT073 in a dose-response manner; 300 pg/ml estradiol mediated a significantly increased colonization compared with unstimulated CFT073 (Figures 6A,C). We also found that estradiol increased the invasion capability of CFT073, at MOI 100, in a dose-dependent manner; 300 pg/ml estradiol mediated a significantly increased invasion of bladder epithelial cells compared with unstimulated CFT073 (Figure 6B).

### Altered Cytokine Release Induced by Estradiol

**FIGURE 7:**
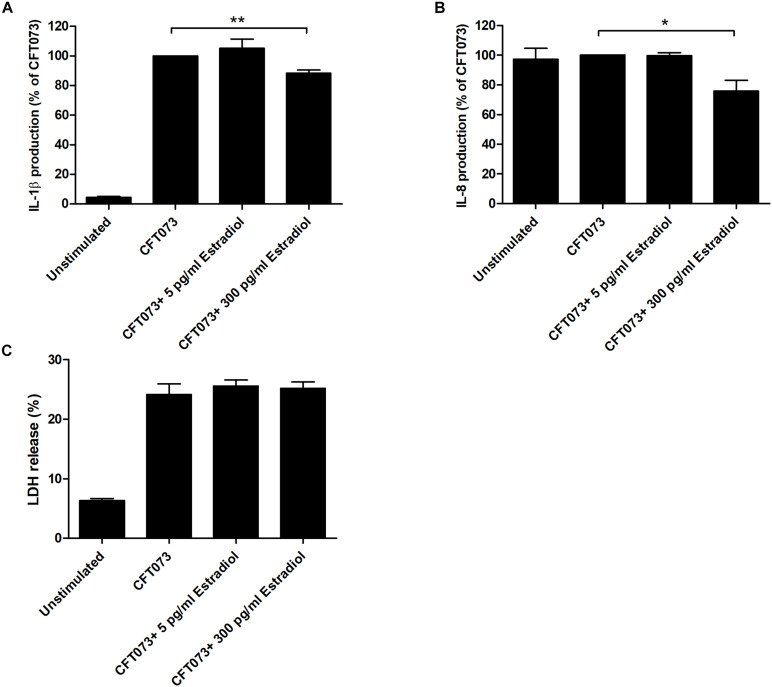
IL-1β **(A)**, IL-8 **(B)**, and LDH **(C)** release from bladder epithelial cells infected with CFT073 pretreated with or without estradiol (5 and 300 pg/ml) at 6 h. Data are presented as mean ± SEM of *n* = 4 independent experiments. The asterisks distinguish statistical significance: *p < 0.05 and **p < 0.01 vs. unstimulated CFT073.

We continued with evaluating if estradiol-stimulated CFT073, at MOI 10, could alter the release of IL-1β and IL-8 from bladder epithelial cells. We found that the cytokine release was significantly lowered by CFT073 stimulated with 300 pg/ml estradiol for both IL-1β and IL-8 compared with unstimulated CFT073 (Figures 7A,B). No difference in IL-1β and IL-8 release was observed for CFT073 stimulated with 5 pg/ml estradiol (Figures 7A,B). Furthermore, we did not find any differences in LDH release from bladder epithelial infected with estradiol-stimulated CFT073 compared with unstimulated CFT073 (Figure 7C).

### Reduced CFT073 Toxicity in the Presence of Estradiol

**FIGURE 8:**
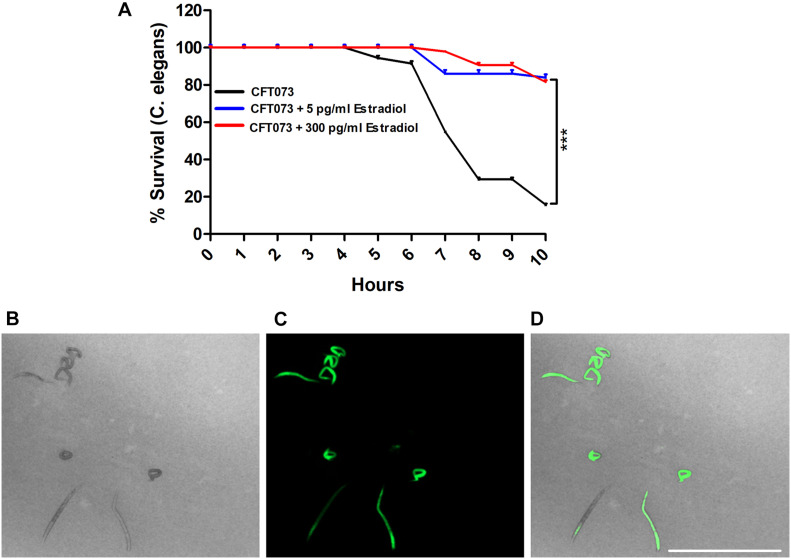
CFT073 mediated *C. elegans* killing in the presence or absence of estradiol (5 and 300 pg/ml) **(A)**. Data are presented as mean ± SEM of *n* = 3 independent experiments. Statistical significance is denoted with asterisks: ***p < 0.001 vs. unstimulated CFT073. Immunofluorescence staining of dead worm with 1 μM SYTOX Green **(B–D)**. Scale bar: 1,000 μm.

We proceeded with evaluating the virulence of CFT073 in the presence of estradiol with an *in vivo C. elegans* infection model. This was done to evaluate the combined significance our findings had on the virulence of CFT073. We found that CFT073 in the presence of 5 and 300 pg/ml estradiol significantly increased the survival of *C. elegans* worms compared with unstimulated CFT073 (Figure 8A). The dead worms were also visualized by the uptake of SYTOX Green (Figures 8B–D). Estradiol *per se* did not induce any *C. elegans* toxicity (data not shown).

## Discussion

Understanding how UPEC interacts and modulates the human host in order to colonize the urinary tract is becoming more important in an age where antibiotic resistance is increasing worldwide. Evidence exists showing that human factors (e.g., cytokines and hormones) synthesized and released by our cells can activate the virulence of different bacteria through bacterial sensors (Mahdavi et al., 2013). The bacterial sensors have a dual function: recognition of the complex host environment and transduction of the message to initiate bacterial adaptation. It is now evident that bacterial virulence is regulated by the detection of host factors released in the microenvironment like hormones and cytokines (Mahdavi et al., 2013; Engelsöy et al., 2019; Gümüş et al., 2019). We know today that estrogen is very important for the protection of the urinary tract against UPEC (Eriksen, 1999; Lüthje et al., 2014). The female body becomes more prone to UTI after menopause because lower estrogen levels lead to lower levels of antimicrobial peptides, vaginal mucosa atrophy, and a higher pH (Gupta et al., 1998; Lüthje et al., 2014). In this study, we focused on investigating the cross-kingdom effects of estradiol on the virulence of UPEC.

Our findings showed that 5 pg/ml of estradiol (postmenopausal concentration) (Stanczyk and Clarke, 2014) significantly increased the growth of the UPEC strain CFT073. However, the premenopausal concentrations of estradiol (100–300 pg/ml) (Stanczyk and Clarke, 2014) did not induce a growth increase. Previous studies have shown that estradiol can increase the growth of several Gram-negative bacteria (Mahdavi et al., 2013; Wang et al., 2013), but we found that this growth increase was associated with postmenopausal, and not premenopausal, concentrations of estradiol. These findings could be a contributing factor explaining why women are more prone to UTI after menopause, as bacterial growth is strongly associated with UTI (Anderson et al., 2004).

We also found that biofilm formation was significantly increased by the postmenopausal concentration of estradiol (5 pg/ml), but not by the premenopausal. Others have also shown that estradiol has the ability to increase the biofilm formation of Gram-negative bacteria (Wang et al., 2013; Fteita et al., 2014). Biofilm formation is associated with protection of UPEC from antimicrobial agents, environmental conditions, and the host immune response (Mah and O’Toole, 2001; Hatt and Rather, 2008). We also investigated if estradiol could alter the expression of UPEC lipopolysaccharide (LPS), which is known to activate and modulate the host immune response during a UTI. However, no differences in the expression of LPS were found in the presence of estradiol. Taken together, we have shown that only the postmenopausal concentration of estradiol induces increased bacterial growth and biofilm formation, which may promote UPEC persistence in the urinary tract of postmenopausal women.

We continued with investigating the effects of estradiol on the metabolic pathways of UPEC. We showed that the gene expression of *pgi* (encoding for glucose-6-phosphate isomerase), a glycolytic enzyme, was decreased by 5 pg/ml estradiol after 6 h and increased after 24 h. We also found that *ppsA* (encoding for phosphoenolpyruvate synthase, an enzyme involved in gluconeogenesis) showed a possible trend toward an increased expression induced by estradiol. Studies have found that tricarboxylic acid cycle (TCA) and gluconeogenesis, but not glycolysis, are essential for the fitness of UPEC in mice (Alteri et al., 2009; Subashchandrabose and Mobley, 2015). We also know that UPEC uses predominantly amino acids and peptides in urine as carbon sources instead of glucose (Alteri et al., 2009). Furthermore, we also found that *frdA* (encoding for the catalytic subunit of fumarate reductase), an enzyme used for anaerobic respiration, was altered and downregulated by estradiol after 6 h. This indicated that aerobic respiration was preferred in the presence of estradiol at an early timepoint. Alteri and Mobley (2015) have previously shown that fumarate reductase is not essential for the fitness of UPEC. Taken together, these data show that estradiol has the ability to modify the expression of various metabolic pathways in UPEC. However, further studies are needed to understand the link between estradiol and the metabolic activity of UPEC.

Iron is a critical nutrient for UPEC survival and pathogenicity in the urinary tract (Subashchandrabose and Mobley, 2015). As soluble iron levels are low in the urinary tract, acquisition of iron is essential for the pathogenicity of UPEC (Roos and Klemm, 2006). Acquisition of iron is facilitated by numerous mechanisms such as outer membrane receptors for heme, ferrous iron transporters, and *via* ferric iron chelators called siderophores (Robinson et al., 2018). We observed that estradiol increased the expression of the salmochelin uptake receptor IroN and the enterobactin uptake receptor Iha (Yep et al., 2014). Upregulation of the uptake receptors may indicate that estradiol alters the iron acquisition systems in UPEC, which may have an effect on UPEC pathogenicity.

The capacity of UPEC to adhere and invade bladder epithelial cells is essential for the colonization of the urinary tract. We found that the gene expression of *fimH* (type-1 fimbriae) and *papC* (P-fimbriae) was upregulated by both 5 and 300 pg/ml estradiol. In addition, we also observed a dose-dependent increased colonization and invasion of bladder epithelial cells mediated by estradiol exposure. If we factor in estrogenic effects on bladder epithelium, it makes these findings even stronger. Premenopausal levels of estrogen are associated with several differences in bladder epithelium compared with lower postmenopausal levels. Higher antimicrobial peptide production, stronger tight junctions between the epithelial cells, and seemingly increased expression of uroplakins and β1-integrins are linked to the higher, premenopausal levels of estrogen (Lüthje et al., 2013). β1-Integrins and uroplakins interact with the type-1 fimbriae adhesin *fimH* and are important for CFT073 invasion into bladder epithelial cells (Eto et al., 2007). Hence, an initial stronger adhesion would lead to increased invasion. In addition, as higher extracellular antimicrobial peptide levels are associated with premenopausal levels of estrogen, the increased colonization and invasion of UPEC at 300 pg/ml could be an antimicrobial peptide evasion strategy.

We continued to investigate if CFT073 exposed to estrogen could alter the cytokine release from bladder epithelial cells. We found that 300 pg/ml estradiol but not 5 pg/ml reduced the ability of CFT073 to induce IL-1β and IL-8 from bladder epithelial cells. This is not an effect of decreased cell viability as no increased LDH release was observed. This very interesting finding supports the hypothesis of host defense avoidance. IL-1β and IL-8 are very important cytokines during a UTI. Both are important for neutrophil recruitment, which is the primary immune cell that clears the infection (Hedges and Svanborg, 1994; Godaly et al., 1997). Taken together, we have shown that 300 pg/ml estradiol increases the colonization of bladder epithelial cells and strengthens the evasion strategies of CFT073.

An *in vivo C. elegans* infection model was used to comprehend how the individual virulence alteration induced by estradiol contributes to the total cytotoxicity of CFT073. We and others have used *C. elegans* previously to evaluate the virulence of UPEC *in vivo*. It was shown that there is a significant correlation between the virulence of UPEC in a murine model and in *C. elegans* (Diard et al., 2007). We showed that CFT073 in the presence of pre- and postmenopausal concentrations of estradiol significantly increased the survival of *C. elegans*. As the estradiol is removed prior to infecting the worms, no alteration in bacterial growth is observed to explain the reduced cytotoxicity. In addition, we did not observe any difference in α-hemolysins activity from CFT073 in the presence of estradiol (data not shown) that could explain our data. However, there are a number of CFT073 virulence factors that we have not explored like the vacuolating autotransporter toxin (Vat) and secreted autotransporter toxin (Sat). Both Vat and Sat have been shown to induce tissue damage (Wiles et al., 2008; Engelsöy et al., 2019). Exploring the effects of estradiol on these toxins may give us a better understanding of why the cytotoxicity of CFT073 is reduced in the presence of estradiol. Taken together, we have shown that estradiol at pre- and postmenopausal concentrations decreases the total cytotoxicity of CFT073.

The limitations of this study include the number of UPEC strains used and the *in vitro* experimental setup. For the results to be generalizable to UPEC in general, an evaluation of other strains than CFT073 is needed. Another limitation of the study is that additional concentrations lower than 5 pg/ml estradiol would be interesting to investigate. A general limitation of the study is that we focus on the direct effects of estradiol on UPEC virulence *in vitro*, without considering the *in vivo* state of multiple host factors and cells interacting with UPEC.

Understanding how the human host affects the virulence of UPEC may be a new frontier in the fight against infection. If we can elucidate how UPEC senses its environment and mobilizes its virulence, we may inhibit this activation and dampen or completely prevent the infection. By focusing of inhibiting virulence, we reduce antibiotic selection pressure (Brannon and Hadjifrangiskou, 2016), which will lead to reduced antibiotic resistance. We have seen that a postmenopausal concentration of estradiol both increased the growth and biofilm formation, which may be a contributing factor as to why women are more prone to UTI after menopause. However, the premenopausal concentration of estradiol mediated the increased bacterial colonization and suppression of proinflammatory cytokines. Our study suggests that the evasion mechanisms induced by UPEC are an adaptation to the primed immune responses associated with high estradiol levels. Although we have found that estradiol can change the virulence of UPEC, further research is needed to understand the mechanism behind these findings and what clinical significance they may have.

## Data Availability Statement

The raw data supporting the conclusions of this article will be made available by the authors, without undue reservation.

## Author Contributions

UE, MS, and ID design the study, analyzed the data, and drafted the article. UE and ID conducted the experiments. All authors read and approved the final manuscript.

## Conflict of Interest

The authors declare that the research was conducted in the absence of any commercial or financial relationships that could be construed as a potential conflict of interest.
